# Changing the game: exploring infants' participation in early play routines

**DOI:** 10.3389/fpsyg.2014.00522

**Published:** 2014-06-06

**Authors:** Valentina Fantasia, Alessandra Fasulo, Alan Costall, Beatriz López

**Affiliations:** Department of Psychology, Centre for Situated Action and Communication, University of PortsmouthPortsmouth, UK

**Keywords:** play, early routine, multimodal interactions, expectations, structured games

## Abstract

Play has proved to have a central role in children's development, most notably in rule learning (Piaget, 1965; Sutton-Smith, 1979) and negotiation of roles and goals (Garvey, 1974; Bruner et al., 1976). Yet very little research has been done on early play. The present study focuses on early social games, i.e., vocal-kinetic play routines that mothers use to interact with infants from very early on. We explored 3-month-old infants and their mothers performing a routine game first in the usual way, then in two violated conditions: without gestures and without sound. The aim of the study is to investigate infants' participation and expectations in the game and whether this participation is affected by changes in the multimodal format of the game. Infants' facial expressions, gaze, and body movements were coded to measure levels of engagement and affective state across the three conditions. Results showed a significant decrease in Limbs Movements and expressions of Positive Affect, an increase in Gaze Away and in Stunned Expression when the game structure was violated. These results indicate that the violated game conditions were experienced as less engaging, either because of an unexpected break in the established joint routine, or simply because they were weaker versions of the same game. Overall, our results suggest that structured, multimodal play routines may constitute interactional contexts that only work as integrated units of auditory and motor resources, representing early communicative contexts which prepare the ground for later, more complex multimodal interactions, such as verbal exchanges.

## Introduction

The present study explores infants' participation in play routines with their mother, through observing their response to un-expected alterations of a familiar social game. Play has been widely explored for its central role in children's development, most notably in rule learning and negotiation of tasks, roles, and goals (Piaget, 1951, 1965; Bruner et al., 1976; Vygotsky, 1978; Sutton-Smith, 1979; Camaioni and Laicardi, 1985). Through play, children learn how to deal with others' expectations and feelings and, even more, they learn about their own feelings, desires and goals when confronted with those of the others (Coleman, 1967; Blurton-Jones, 1976). Playing together is not merely the sum of single responses to the partner's play, but rather a creative process emerging from the interactional dynamics between different individuals in a specific cultural context (Fogel, 1993). To be engaged in a social game is thus—at the same time—a developmental goal and an instrument through which development occurs, and an ideal place to investigate social interactions. Although it is generally agreed in developmental research that children's social play has intrinsic co-operative qualities, the same has not been demonstrated for infants' play. Indeed, relatively little research has been done on the formats and structures of early mother-infant multimodal games, and how infants participate in them.

Previous studies have explored the mother-infant's mutual building up of “free” play interactions around 3 months of age (Stern, 1974; Trevarthen, 1988) and spontaneous *peekaboo* play episodes in the context of language acquisition from around 5 months (Bruner and Sherwood, 1976; Ratner and Bruner, 1977). Mother-infant free play has been also described in terms of negotiation of interactional boundaries and points of transition (Stern, 1974), the different ways of alternating participation (turn-taking) and the manners in which a play sequence may be built up through a considerable spatio-temporal structuring (Garvey, 1974). Besides these pioneering studies, very little research has been done on structured game routines in infancy, like early nursery rhymes or vocal-kinetic combinations of gestures and songs (Mehus, 2011). Since these routines are well known to be played by mothers from very early on, they are also part of the infant's daily experience of participating in structured, meaningful interactions, such as being fed or dressed up, or play a familiar game. These routines have proved to help infants to coordinate with the adult's actions (Trevarthen, 1979; Hubley, 1983) and to become skilled cooperative agents as they participate in them (Lerner et al., 2011; Rączaszek-Leonardi et al., 2013). Routines, as they are familiar and predictable, also orient the infant's capacity to anticipate the other's action and create expectations on the other's behavior (Ambrosini et al., 2013; Reddy et al., 2013).

Infants' expectations have been an area of considerable investigation in developmental research (Spelke, 1985; Baillargeon, 1994). One way to explore them is by introducing changes in a familiar situation, that is, by violating expectations. Studies using this method have typically focused on the infant's reaction to maternal breach in engagement, or withdrawal from the ongoing interaction. Research showed that at around 3 months of age infants react by frowning and gazing away from an adult who abruptly stopped interacting with them (Lamb et al., 1987; Tronick and Cohn, 1989). At around 4–5 months infants protest (crying more loudly) and orient away when an adult intentionally fails to soothe them by picking up, letting the infants' expectations unmet (Lamb and Malkin, 1986); around 9 months infants can detect game interruptions by their playing partner, increasing their vocalizations to call her back in the game (Ross and Lollis, 1987), and at 10 months they increase their gaze to an adult's face whose action was blocking the infant's play with a toy (Phillips et al., 1992). These studies support the idea that infants are sensitive to alterations of the adult's usual behavior from very early on^1^.

Our study differs from previous research, as it looks at the infant's reaction to violations of the multimodal format of a familiar play routine by a partner that is still affectively engaged with her. Notwithstanding, it shares the same conceptual grounding of previous research: observing the infants' participation in a familiar situation and the way it changes in response to unexpected behaviors, in order to learn more about how infants take part in, and make sense of early social interactions. The aim of the present study is thus to explore the structure of early social games commonly played by mothers, and how the infants participate in them. In addition to this, we want to investigate whether infants show signs of expectations on the game structure by looking at how their participation changes if the familiar game is played differently. To do this, we observed twenty 3-month-old infants and mothers playing a structured, multimodal game in the lab as they usually do at home, and subsequently a unimodal, violated version of the same game: without gestures and without sounds. Limbs Movements, Gaze Away and facial expressions were coded and compared across conditions, to measure changes in the infants' behavior as response to violations of the game as expected.

## Materials and method

### Participants

Twenty mothers and their 3-month-old infants (10 girls, 10 boys) (*M* = 96 days; *SD* = 4.04 days) participated in the study. All the mothers have been living in the UK since at least 10 years, and two mothers out of 20 were not native British citizens. All infants were Caucasian and on average healthy birth weight (*M* = 3.36 Kg; *SD* = 0.40 Kg). Seven infants were firstborns, four were born with slight complications, three mothers underwent a C-section and one mother had a particularly long labor. The mothers' ages at the time of birth ranged from 26 to 37 years (*M* = 31 years, *SD* = 3.44). Five dyads were excluded from the original sample of 25 infants due to the infants' fussiness and lack of interest at the beginning or in the middle of the procedure. Volunteer parents were recruited through different children and family centers, nurseries and pre/antenatal classes in town, which resulted in an heterogeneous socioeconomic background.

The *Bayley Scales of Infant Development*—Second Edition (BSID-II) (Bayley, 2006) were used to check the infants' motor maturity, cognitive skills, and developmental age equivalent. Results from the Bayley Mental and Motor Scales assessment showed that only one out of twenty infants scored lower than one percentile under the average in the Mental Scale (Mental Index score = 82) but not in the Motor Scale (Motor Index score = 88). This baby's behavioral responses were checked and resulted as not performing distinctively different from the average responses of the other infants. Thus, this one baby was not removed from the sample. Results are shown in Table 1.

**Table 1:** **Bayley scores and age equivalents**.

**Measure**	**Mean index score**	***SD***	**Mean developmental age equivalent**
Mental scale	88.5^a^	12.1	2.8 Months
Motor scale	92.8^b^	7.8	3 Months

a *Mental score range: 82–104*.

b *Motor score range: 88–105*.

### Procedure

Mother-infant dyads were observed in a quiet, spacious room, and to avoid any additional stress observations were arranged at a convenient time for mothers. All of the procedures in the study underwent ethical approval by the Science Faculty Ethics Committee, which abides by the BPS Guidelines for Research with Human Participants, and all the mothers were asked to sign a written informed consent.

The observation room contained a soft mat placed on the floor with some toys, a table with two chairs and four sofas. The experimenter was helped by an assistant who, at the beginning of the observation, asked the mother general information about the infant and the kind of games they usually play together. Before administering the BSID the experimenter and the infant played on the mat to get familiar for approximately 3–5 min. The length of BSID assessment was on average 12 min. Then the play observation began, consisting of three phases: an initial warming up period of approximately 5–7 min, a “normal” performance of a familiar game (normal condition) and then two variations of that same game (no-sound and no-gestures conditions).

Since our specific interest was to explore whether changes in the multimodal elements composing the game format affect infants' participation in the game, we focused on violations which do not expose the infant to a maternal withdrawal of engagement. In the normal condition phase, our baseline episode, mothers were asked to play one or two routine social games, of the kind of nurseries rhymes, in the same way she would normally do at home. As described above, these kind of social games have a vocal-kinetic format, as they are compound by a (usually) rhymed song accompanied by hand gestures. To investigate the infants' participation and expectations on the game structure, mothers were asked to perform the same game in two variants: once without using any sound (no-sound condition) and once without doing any gestures (no-gestures condition). Namely, mothers made no movements in the no-gesture condition, and made no sounds in the no-sound condition. The performance of each condition was spaced out by approximately 2 min of free interaction, and the violated conditions sequence was randomized between and within infants to control for order effect.

Mothers were not instructed to avoid any particular affective behavior (i.e., to display a neutral face, or avoid smiling, or looking at the infant), but encouraged to hold the baby in the same position she had done during the normal condition also when she had to make no gestures. For instance, if the position of the infant was to be held up by the armpit in the normal version of the game, we asked to mother to keep holding the baby in the same way in all the three conditions. In the no-gestures condition the mother was asked not to move her hands or the baby (shaking, pulling, bouncing up, and so on), whatever her position. If the infant gave signs of distraction or discomfort, the procedure was stopped and resumed from the last game condition (when possible); this happened three times out of 20 infants, spread over conditions.

The entire sequence was videotaped by two cameras mounted on tripods. One camera was positioned on a 45° angle from the mother, triadically with the camera and the infant; the other camera was fixed focusing on the mat to be used for the BSID assessment.

### Coding

The infants' Limbs Movements, gaze, and facial expressions were coded from video recordings of the entire procedure. These measures have been widely used in the literature on infants' social expectations (Toda and Fogel, 1993; Legerstee and Markova, 2007). For instance, attention patterns like gaze orientation are revealing of infants' emotions. Infants have been shown to look intently at interesting stimuli, but to avert gaze from a person who stare at them impassively (Tronick et al., 1978; Toda and Fogel, 1993). Body movements have been found to be powerful indicators of infants' discrimination of the other's intentional vs. unintentional actions (Behne et al., 2005), and infants' anticipatory adjustments during picking up (Service, 1984; Reddy et al., 2013). As Adolph and Berger wrote (2006), “Movement is perhaps the most ubiquitous, pervasive, and fundamental of all psychological activity. It is the hallmark of animacy and the essence of agency” (p. 181). The relative frequency of presence/absence of each behavior was coded second-by-second, and only once for each second by a coder blind to the experimental hypotheses and conditions. For the coding we used ELAN, a video analysis software that allows for the creation of complex annotations on video and audio resources (Wittenburg et al., 2006). A second blind observer independently coded 50% of the infants (10 infants in all three conditions). Inter-observer agreement was determined by using Cohen's Kappa coefficient. Reliability was high for all behaviors (Positive Affects κ = 0.82), Negative Affects, κ = 0.75, Gaze Away, κ = 0.78, Limbs Movements, κ = 0.85; all *p* < 0.001.

#### Limbs movements

Limbs Movements are the combined coding of arms and legs A code of leg or arm movement was assigned when there was a substantial change of position in space observed in arms or legs. Shivers, trembling, or jerky moves were not considered as movements.

#### Gaze away

The infants' gaze was coded as “Away” every time the infant looked sideways, up to the ceiling or when the infant's head was turned off from the mother's face.

#### Positive and negative affect

Infants' facial expressions were coded as “Positive” and “Negative” Affects (Camras and Shutter, 2010). Positive Affect was encoded as smiles (raised cheeks and corner of lips turned up with mouth open or closed) and laughs (raised cheeks, mouth open, lower and upper gum visible, eyes open or winked, possibly accompanied by some vocalizations). A code of Negative Affect was assigned to frowns (furrowed brow and downturned mouth) and sad expressions (mouth, eye brows, and cheeks turned down) (Legerstee and Markova, 2007).

#### Stunned expression

A coding of Stunned Expression was assigned when the infant showed wide open eyes, open mouth or mouth close but still, neutral lips (Meltzoff and Moore, 1977). Previous studies on violations of expectations have used “puzzlement” as dependent variable as index of the infant's reaction to ambiguous and unexpected stimuli (Tronick, 1989; Camras et al., 2002). With respect to stunned expressions though, puzzlement seems a less neutral measure. So, we decided to code components of puzzlement such as eyebrow frowning and downturned lips as Negative Affect; instead, with Stunned Expression we wanted to capture as widely as possible any infants' reaction of surprise and uncertainty.

### Games durations and selection

We asked the mothers to play a routine game which included singing a song and gesturing, that was also familiar for the infant. When dyads had more than one type of game recorded, we used the game that was more familiar for the infant according to what mothers told us in the preliminary interview. When played normally, games lasted approximately 28 s (*M* = 28.04 s; *SD* = 0.24); when violations were introduced, games mostly maintained their original lengths (no-gestures: *M* = 27.9 s; *SD* = 1.4 s; no-sound: *M* = 27.5 s; *SD* = 1.2 s). A Friedman's analysis of variance (ANOVA) was performed to control that game durations within babies had not significantly changed when the mothers introduced the two violations. Tests were conducted using Bonferroni adjusted alpha levels of 0.02 (0.05/3). Results confirmed that games lengths across conditions did not significantly differ [χ^2^_(2)_ = 2.784, *p* = 0.249], and therefore games lengths have not been standardized.

### Data analyses

Because of nature and quality of data (frequencies), the small sample size and repeated observations, non-parametric repeated measures analyses were performed. Tests were conducted using Bonferroni correction of 0.01 per test (0.05/5), and were exact and two tailed. Friedman's ANOVA has been used to compare infants' Limbs Movements, Gaze Away and affective expressions across conditions (normal, no-gestures, and no-sound). ANOVA results were followed-up by pairwise comparisons between conditions using Wilcoxon Signed Ranks test. A nonparametric measure of the effect size, *r*, was used (Ivarsson et al., 2013) and results are showed in the following section. No significant effect of the order in which the two violated conditions were presented was found.

## Results

### Games description

The dyads we observed played games which present similar formats, with few structural differences. They are built on units of sequenced actions formed by patterns of gestures and vocalizations often repeated throughout the game. Games differed in their basic features, i.e., rhythm, type of gesturing, voice tone, the position of the infant and the mother (see Table 2). Some games had the infant upright seating with arms held forward by the mother, others had the infant laying on the back; few games had the infant made flying up and down held on the waist by the mother. Similar games appeared to have been adapted by mothers and showed some variations, mainly in the infant's posture. For example “*Row Row the Boat*,” in which the mother performs a rowing motion so that the infant repeatedly leans toward the mother and away, was played in three different variants: with the infant's upright seated, or laying on the mat or embedded in to the mother's stretched legs. Overall, the games appeared to be well tuned on to the infants' attentional abilities, alternating patterns of increasing stimulation (e.g., higher pitch of voice, faster movements) with periods of decreasing activity and pauses. Furthermore, their structure was build up on repetitions and rhymes. In “*Head, Shoulder, Knees and Toes*,” for instance, the refrain was symmetrically placed at the beginning and the end of the game, as opening and closure, but sung with a different intonation.

**Table 2:** **Types and structure of mother-infant early social games**.

Name of the game	*Row Row the Boat*	*Head, Shoulder, Knees*	*Hickory Dickory Dock*	*The Grand Old Duke of York*	Other games
Number of dyads	8	4	3	3	2
Position of the infant.	Usually seated.	Laying down.	Laying down.	Laying down or held up.	Laying down.
Position of the mother.	Seated facing the infant.	Leaning forward upon the infant, rarely seated.	Leaning forward upon the infant or seated.	Leaning forward or steadily seated.	Leaning forward.
Structure	The mother is holding the baby by the arms performing a rowing motion with them, swinging the infant back and forth repeatedly. The song is divided in lines. Each line has a peak of intonation in the middle, and a pause at the end before the next line starts.	The mother touches the baby's bodily part as she is naming them in the song, starting from the head. The first verse of the song is repeated twice at the beginning and closure of the game with a different intonation. The song has a peak of vocal tension and acceleration in the middle, which then slowly decreases until the end of the game.	The mother alternates between touching the infant's body parts and clapping her own hands. In the ned she holds the baby's legs up, swinging them sideway. The song has a peak of vocal tension and acceleration in the middle, which then slowly decreases until the end of the game. The main line (Hickory Dickory Dock) is repeated in the end.	One version has the mother holding the baby's hands moving them up and down. Another version has the mother holding the baby up to make her rocking up and down. The song accompanies all the game through, and accelerates until it reaches a peak of intonation toward the end, which quickly drops in the end.	The mother kissed the baby's cheek while gently looming. In one other game the mother shook up the baby's legs making a loud sound and singing a song. In both the games a song accompanies the gesturing, alternating peaks of intonation and accelerations with pauses and decelerations.

**Figure 1:**
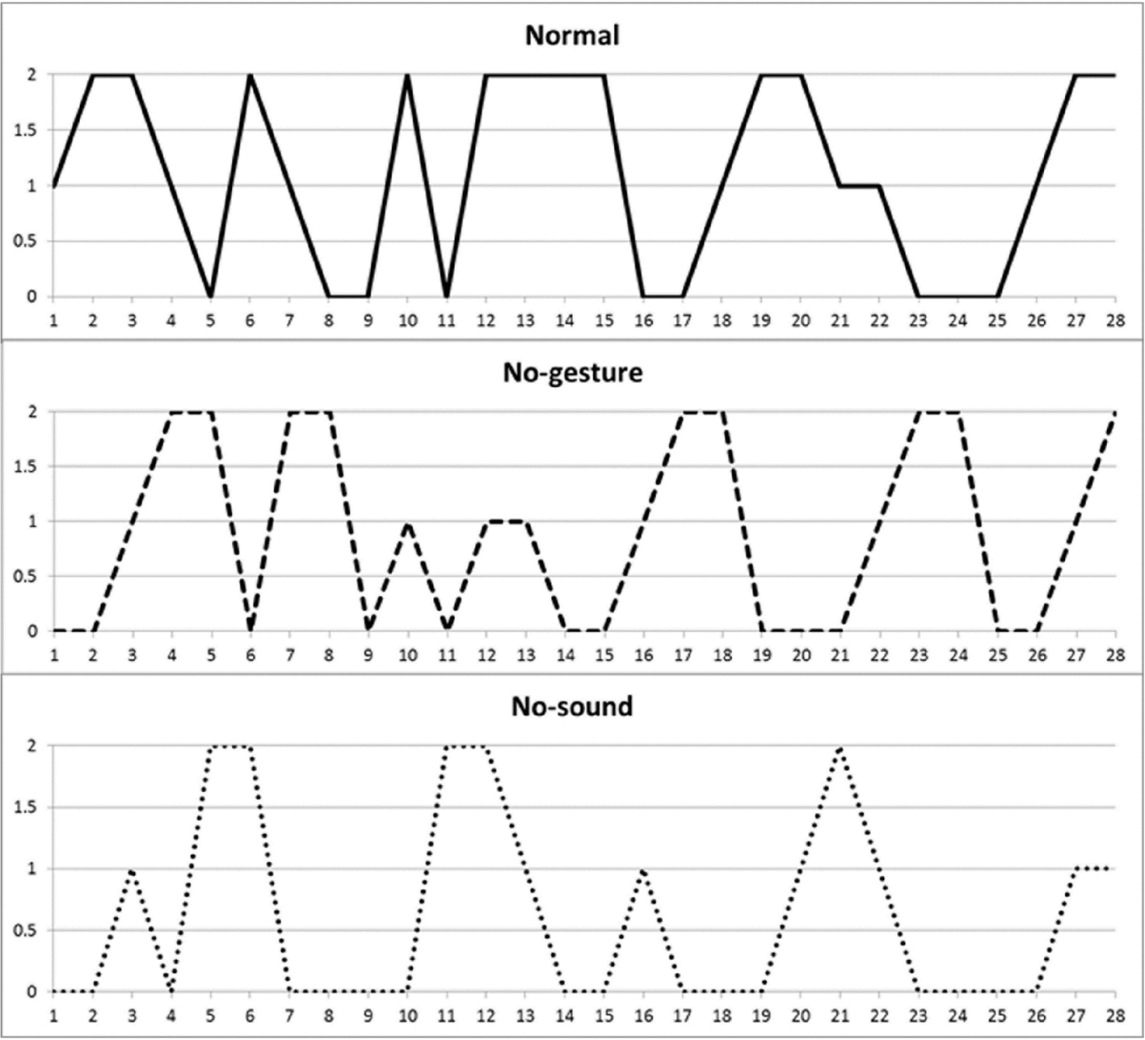
**Individual Limbs Movements of R. playing *Row Row the Boat* in the three conditions**. The order of condition presented was normal, no-gesture, and no-sound. A score of 2 indicates that movements of both the arms and the legs simultaneously, whereas a score of 1 indicate a single movement of either the arms or the legs. A score of 0 indicate absence of movement.

**Figure 2:**
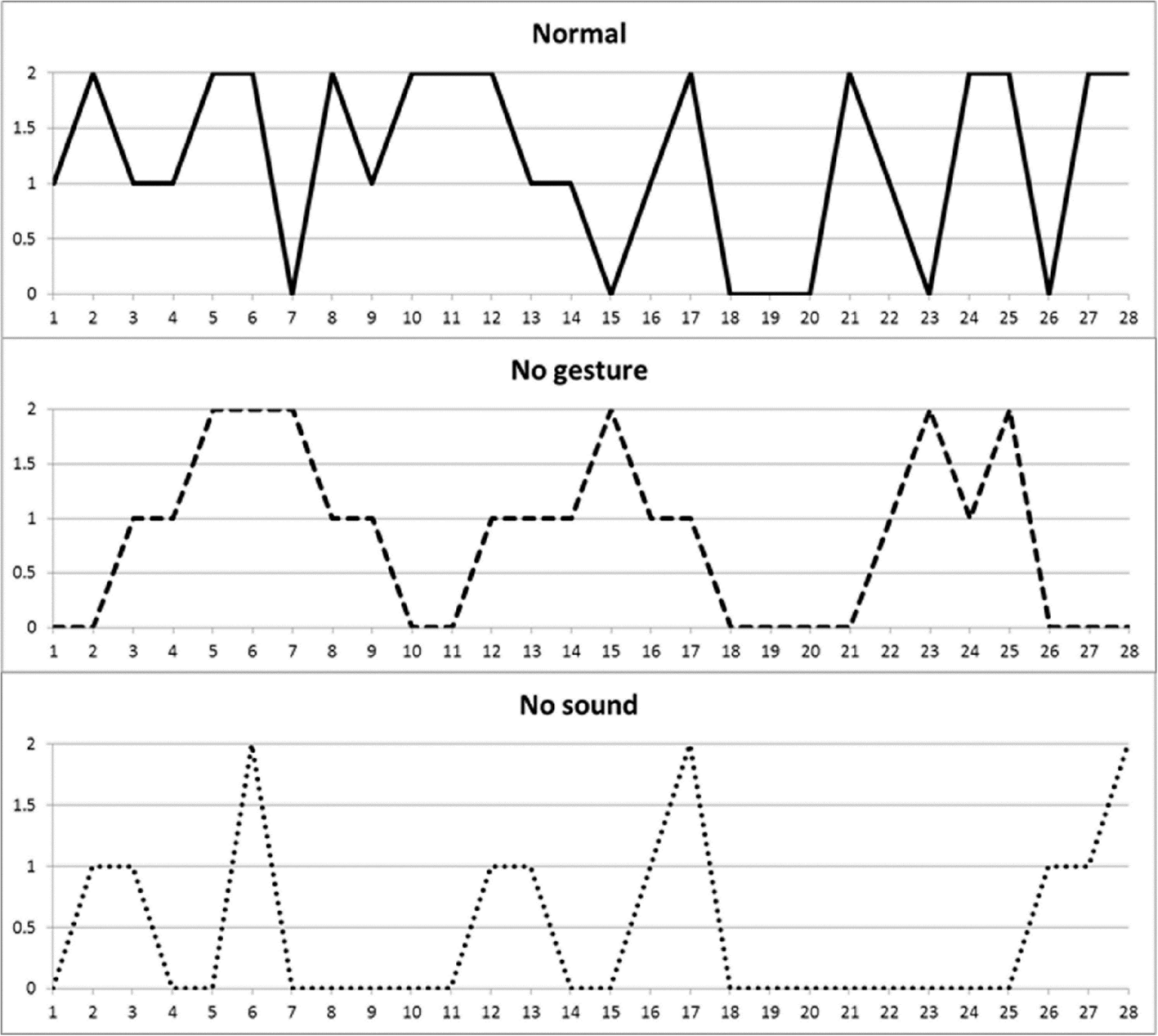
**Individual Limbs Movements of K. playing *Head, Shoulder, Knees, and Toes* in the three conditions**. The order of condition presented was normal, no-sound and no-gesture. A score of 2 indicates that movements of both the arms and the legs simultaneously, whereas a score of 1 indicate a single movement of either the arms or the legs. A score of 0 indicate absence of movement.

Qualitative observations of the videos also revealed similar individual patterns of movements in infants playing the same kind of games in the normal conditions. For instance, infants playing “*Row Row the Boat”* showed similar frequencies of Limbs Movements: higher within the first 5 s of the game (*M* = 4, *SD* = 0.07), decreasing in the middle (approximately after 10–12 s; *M* = 3.25, *SD* = 0.66) and lower in the last 5 s of the game (*M* = 3.11, *SD* = 0.57). On the contrary, infants playing “*Head, Shoulder, Knees and Toes”* moved both the arms and legs more in the middle of the game (approximately after 9–10 s; *M* = 4.67, *SD* = 0.047) compared to the first 5 s of the game (*M* = 3.33, *SD* = 0.47) and the last 5 s (*M* = 2.33, *SD* = 0.47). Examples of two infants' individual bodily patterns are shown in Figures 1, 2. Higher scores represent movements of both the arms and the legs simultaneously, whereas lower scores represent single movements either of the arms or the legs or absence of movements.

Figure 1 represents individual patterns of one infant (R.). The first peak of arms and legs movements appears after about 5 s from the beginning in the normal condition, but only after about 6 s in the no-sound. Similarly, another peak of movements is shown after approximately 12 s when the game is played normally, only to appear with almost 1 s later in both the violated conditions. Furthermore, the infant moves from the beginning to the completion of the routine, i.e., form the first to the last second, only when the game is played normally and with gestures, but he starts moving 1 s later in the no sound condition. Similar results were found in 12 infants out of 20, showing that 6 infants delayed their movements only in the no sound condition, 4 infants delayed their movements in both the no-sound and no-gestures conditions and 2 infants only in the no-gestures condition.

Figure 2 depicts another infant (K.) during “*Head, Shoulder, Knees and Toes*.” When the game is played normally, K. moves more and for longer periods than in the no-sound or no-gesture conditions. She also holds longer periods of arms and legs stillness when the game has no sound compared to its normal version, with movements eventually fading out as the game comes to the end. Since K appears to be overall very active when the game is played normally, moving frequently and often combining both arms and legs, her periods of stillness in both the no-sound and no-gestures conditions stand out even more evidently than in R.

**Figure 3:**
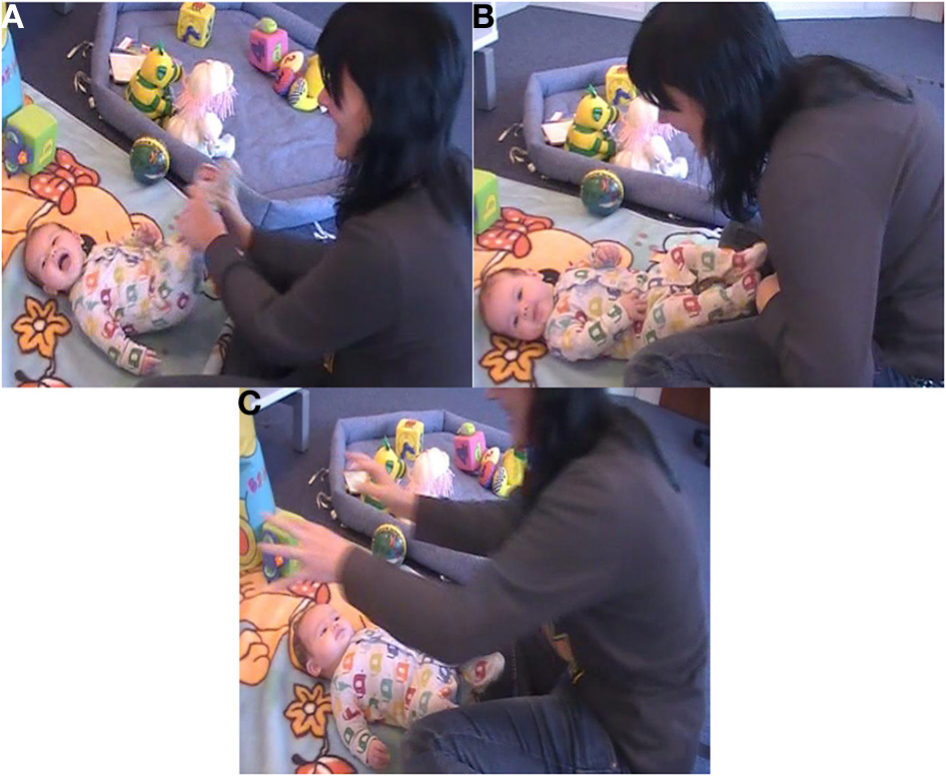
**R. playing *Hickory Dickory Dock* normally (A), with no gestures (B), and no sound (C)**.

Figure 3 shows one play interactions and how the infant's participation changed when the game was altered. In Figure 3A we see baby R. playing “*Hickory, Dickory Dock*.” During the normal version of the game, R. is openly laughing, and his upper body seems slightly twisted, as to accompany the mother's movement. He vocalizes vividly, and seems enjoying the play interaction. In Figure 3B R. is again looking away, but he does not show signs of enjoyment. His arms and legs are still and relaxed, and he seems not focused on the game but attending to something external, behind the camera. Finally, in Figure 3C R. appears very concentrated on the mother's action, but not affectively participating: he does not show any positive affective expression, and he seems quite bodily still.

### Effect of games violations on the infants' behavior

Mean and standard deviation values of the infants' behavioral responses are presented in Table 3. Analyses of Friedman's ANOVA were conducted for each of the dependent measures, revealing significant effect of game violations on Limbs Movements χ^2^_(2)_ = 27.410, *p* < 0.001, Gaze Away, χ^2^_(2)_ = 13.914, *p* = 0.001, Positive Affect, χ^2^_(2)_ = 29.059, *p* < 0.001, and Stunned Expression, χ^2^_(2)_ = 8.044, *p* = 0.001. No significant differences were found for Negative Affect, χ^2^_(2)_ = 5.344, *p* = 0.069. The Wilcoxon Signed-Rank test showed that Limbs Movements were significantly higher in the normal compared to the no-sound (*z* = −3.923, *p* < 0.001, *r* = 0.877) and no-gestures (*z* = −3.728, *p* < 0.001, *r* = 0.877) conditions. According to Cohen (1988), the effect of these differences was large in both cases. No differences were found between the two violated conditions (*z* = −1.192, *p* = 0.233). Gaze Away comparisons showed that infants gazed away more often in the no-sound than the normal (*z* = −3.626, *p* < 0.001, *r* = 0.468), and no-gestures condition (*z* = −2.600, *p* = 0.009, *r* = 0.335.) but not in the no-gestures compared to normal condition (*z* = −1.462, *p* = 0.144). Positive Affect was significantly higher in the normal condition than the no-sound (*z* = −3.652, *p* < 0.001, *r* = 0.471) and no-gestures (*z* = −2.883, *p* = 0.004, *r* = 0.372), and significantly higher in the no-gestures condition than the no-sound (*z* = −3.823, *p* < 0.001, *r* = 0.493). Results also showed that infants had significantly more Stunned Expressions in the no-sound compared to the normal condition (*z* = −2.546, *p* = 0.001, *r* = 0.328), and in the no-sound compared to no-gestures (*z* = −3.453, *p* = 0.001, *r* = 0.445). No significant increase in Stunned Expression was found in the no-gesture (*z* = −0.577, *p* = 0.564) compared to the normal condition.

**Table 3:** **Means and standard deviations of relative behavioral frequencies**.

**Behavioral measure**	**Normal**		**No-gestures**		**No-sound**	
	***M***	***SD***	***M***	***SD***	***M***	***SD***
Limbs Movements	25.9	9.49	18.20	7.17	15.01	6.04
Gaze Away	1.20	0.96	1.65	0.89	2.65	1.08
Positive Affect	2.85	1.31	1.9	0.77	0.75	0.62
Negative Affect	0.5	0.29	0.75	0.34	1.1	0.39
Stunned Expression	0.21	0.09	0.35	0.12	0.80	0.28

*The maximum duration of the games in every condition was 30 s, thus each behavioral measure may range from 0 to 30*.

## Discussion

The present study explored early play routines of 3-month-old infants and their mothers, and observed the infant's behavior when these routines were disrupted. As there is very little literature about early play routines, our aim was firstly to describe them in order to understand their structure, and what kind of participatory affordances do they offer to infants.

In addition to this we wanted to observe whether the infants' participatory behavior would change, as a result of unexpected alterations of the game. Our analyses showed that when the game was violated with sound and gesturing disjointed (in both the no-gestures and no-sound conditions), the majority of infants significantly decreased their movements, gazed away from the mother more often and decreased their positive affect display. Furthermore, they presented increased Stunned Expressions, especially when the game was played without any sound. Overall, we can argue that the infant's participation in the game was poorer in the altered conditions. A possible explanation for this may be that infants are more likely to experience interactions that are coordinated at the motor-auditory level, involving sounds without gestures, rather than gesturing movements not accompanied by any vocalization (Mehus, 2011). Yet, these findings may lead to different interpretations. On the one hand, violations may have not been recognized as such, but simply experienced as different, less engaging activities than the fully enacted games. Or they might have become tired or bored as the procedure went on, and therefore engaged less. Support for this interpretation comes from absence of any signs of distress (in terms of Negative Affect), as typical when expectations are violated, and presence of some signs of inattention (such as Gaze Away). Yet, this would not explain why they showed more Stunned Expressions.

An alternative interpretation which may be advanced is that infants' decreased their participation by smiling and laughing less, showing increased Stunned Expressions and being more bodily still, as the result of confusion for something ambiguous that did not match their usual experience (Tronick et al., 1978). According to this interpretation, infants may have developed expectations regarding how the mother usually behaves in such specific interactions, which in turn affected the quality of their participation when the familiar game was violated. We support this second alternative. The most persuasive evidence for it is infants' dramatic behavioral change in the altered conditions even if the mother had not withdrawn from the interaction and was still offering some level of stimulation. This represents a point of difference with most of the research using violation paradigm, in which the adult interrupts an initiated interaction or strongly reduces her interactional engagement (by suspending the gesturing or singing). A weakened engagement in the game and—even more importantly—the loss of its playful quality, as shown by the decrease in positive affect, might mean that the infants were not so much affected by a lack of maternal contingency or affective attunement, (as observed in many contingency violation studies^1^) but rather by alterations of an established game structure. If this interpretation is correct, play routines may constitute early interactional contexts on which infants have expectations as structured units of coupled auditory and motor resources.

Observing the games structures, we found that they provide the infants with multiple opportunities of engaging in the interaction. Furthermore, they seem to represent a “ready-to-use,” interactive tool for parents. We think that compared to free play, these structures enable and sustain the infant's participation in the interaction for long periods, supporting the development of interactive competences of reading complex communications. Surprisingly, the games we observed presented similar lengths and format even if their structure differed, suggesting that they may respond to specific developmental needs: to be entertaining for the infant, to facilitate an affective and pleasant experience between the infant and the mother, and have a flexible structure to be adapted to the baby's emerging capacities (cognitive, attentional, motor capacities). As routines, these games may also have a developmental function: conceiving a routine, whatever that might be, as a sequence of recognizable tasks-so-far (Lerner et al., 2011) enables its understanding as based on a situated, practical grasp of that routine instead of relying on some cognitive representation of it. In other words, it may enable infants of being capable partners in joint actions (as they recognize and have expectations on it) even without possessing higher-level social knowledge. Under this view, infants are not passive recipients of actions performed on them, but rather capable of active participation in any joint routine. Play routines might also represent early communicative contexts which prepare the ground for later, more complex multimodal interactions, such as verbal exchanges (Bruner, 1975; Bullowa, 1979). As Goodwin (2013) proposed, interactions. As Goodwin (2013) proposed, interactions are co-operative and transformative in the sense that “Actors can build new action by selectively reusing resources provided by a prior action” (p. 1), suggesting that if interactions are constructed out of different resources then even non-verbal participants may co-contribute to the building up of an interaction. Multimodality can be therefore framed as structuring and facilitating early interactions through co-participation.

Whatever interpretation we support, this study has led us to reflect about how an embodied participation in joint routines generates expectations on the partners' mutual commitment to participate in a certain—though not identical—way. The pleasure of participating seems at least partially conditional to recognizing the moves in the sequence and being therefore able to cooperate to and in it. Since this is the first work to explore early structured play, it also presents various limitations. For instance, to endorse an ecological approach and explore how early social games worked in the first place, we decided not to constrain mothers to play a specific game but rather focus on their spontaneous way of playing. This is why only the two violated conditions have been counterbalanced, but not the normal one. We preferred to start always with the normal game to preserve (and grasp) the infant's spontaneous engagement with a familiar routine, which would not be possible if we started with the other conditions.

### Conflict of interest statement

The authors declare that the research was conducted in the absence of any commercial or financial relationships that could be construed as a potential conflict of interest.

## Footnotes

^1^See Murray and Trevarthen (1986) and Nadel et al. (1999) for contingency expectancies studies; see also Tronick et al. (1978), for still face paradigm studies.
